# The Draining Lymph Node in Rheumatoid Arthritis: Current Concepts and Research Perspectives

**DOI:** 10.1155/2015/420251

**Published:** 2015-02-22

**Authors:** Francesca Benaglio, Barbara Vitolo, Martina Scarabelli, Elisa Binda, Serena Bugatti, Roberto Caporali, Carlomaurizio Montecucco, Antonio Manzo

**Affiliations:** Rheumatology and Translational Immunology Research Laboratories (LaRIT), Division of Rheumatology, IRCCS Policlinico S. Matteo Foundation/University of Pavia, Piazzale Golgi 2, 27100 Pavia, Italy

## Abstract

Rheumatoid arthritis (RA) is a chronic autoimmune inflammatory disease of unknown aetiology, leading to progressive damage of bone and cartilage with functional impairment and disability. Whilst the synovial membrane represents the epicentre of the immune-inflammatory process, there is growing evidence indicating the potential involvement of additional anatomical compartments, such as the lung, bone marrow, and secondary lymphoid tissues. Draining lymph nodes represent the elective site for tissue immune-surveillance, for the generation of adaptive immune responses and a candidate compartment for the maintenance of peripheral tolerance. Despite the precise role of the juxta- and extra-articular lymph node stations in the pathogenesis of RA remaining poorly defined, several lines of research exploiting new technological approaches are now focusing on their assessment as a potential new source of pathobiologic information, biomarkers, and complementary therapeutic targets. In this review we present an updated overview of the main concepts driving lymph node research in RA, highlighting the most relevant findings, current hypothesis, and translational perspectives.

## 1. Introduction

In chronic inflammatory and autoimmune diseases, the cross-talk and reciprocal modulation between peripheral and lymphoid tissues represent an integral part in the development, maintenance and progression of undesired immune responses. Studies using the nonobese diabetic (NOD) mouse model of autoimmune diabetes have shown that islet-cell antigens are presented to autoreactive T cells within the pancreatic draining lymph nodes (LNs) [1], and early LN excision protects mice against insulin autoantibodies, insulitis, and diabetes development [2]. Upon initiation of inflammation, cellular and molecular trafficking between the periphery and the draining LNs through the expanded lymphatic vascular network significantly influences the progression of the inflammatory response by modulating the degree of tissue infiltration and shaping the immune response [3–5]. Recent experimental studies in animal models have indeed shown that pharmacological manipulation of the peripheral tissue-LN connection might represent a novel therapeutic strategy for inflammatory disorders [6–8]. Altogether, the above observations indicate that peripheral tissues and draining LNs operate as a functional unit during chronic inflammation, and full understanding of how local pathological processes originate and progress cannot disregard the analysis of the tight interconnection and reciprocal modulation between the two compartments.

Rheumatoid arthritis (RA) is a complex autoimmune disorder that primarily targets the synovium of diarthrodial joints. Cell-cell and cytokine networks established within the inflamed RA synovium sustain disease chronicity, amplify autoimmune responses and ultimately elicit cartilage and bone destruction [9–11]. Synovial pathobiology has offered crucial guidance for the development of efficacious therapeutics and still remains a rich source of investigation for the advancement in the biological and clinical understanding of RA [12–14]. Consistent data have however now established that the synovial compartment is neither the initiator nor the sole player of RA. The identification that systemic autoimmunity may antedate the onset of clinical arthritis by several years [15, 16], and synovial involvement does not appear prominent until the later stages of disease development [17–20], has redirect interest towards possible extra-articular sites of origin of RA-associated immunological disturbances, such as mucous membrane sites of the respiratory and gastroenteric tract as well as the oral cavity [21–24]. Furthermore, despite synovial involvement remains the cardinal feature of clinical RA, other joint compartments appear at least as affected in course of the disease. The subchondral bone marrow adjacent to inflamed joints indeed undergoes inflammatory changes since the earliest phases of RA [25, 26], and marrow inflammation appears intimately linked with synovitis and subsequent pathologic bone remodeling [27, 28].

Collectively, compared to organ-specific autoimmune conditions, in which the autoimmune reaction targets a particular organ via a humoral and/or cellular response against specific autoantigens since the very early phases of the disease, the pathogenic processes of RA thus appear to originate and progress in a variety of anatomical districts. Such complexity, together with the lack of knowledge of the target autoantigen(s), makes the analysis of local and systemic lymphoid tissue reactions particularly challenging in RA. Experimental animal models of arthritis and the introduction of imaging techniques capable of evaluating LN responses in humans have only recently opened a window onto the possible participation of the lymphoid compartment to RA. In this review, we present an updated summary of the main findings related to LN involvement and potential pathobiologic role in course of chronic arthritis. To provide a contextualized framework of the anatomical concepts, functional processes and imaging findings that have been the object of analysis in pathological settings, the review is introduced by a brief summary of the general structural features of the homeostatic LN and of the main structural changes induced by immunological challenges under controlled experimental conditions.

## 2. Lymph Node Homeostatic Structure

LN are encapsulated oval-shaped fibrovascular organs, strategically distributed through the body of mammals, that represent the elective site for lymph filtration, antigen surveillance, antigen-presenting cell (APC)-lymphocyte interactions, effector/memory cell generation and antibody secretion. These functions are favored by the dynamic structure of the LN, capable of integrating cell trafficking from the bloodstream, drainage of cells and soluble factors from peripheral tissues through the afferent lymphatic system and effector cell/molecule output from the efferent lymph.

The anatomical and functional nodal unit is the lymphoid lobule, a polarized structure that can be schematically subdivided into three main areas with specific and complementary roles: (1) an apex, consisting of the B cell rich superficial cortex, where B lymphocytes can interact with resident follicular dendritic cells (FDCs) and undergo affinity maturation within germinal centres (GCs), (2) a deep paracortex, characterized by paracortical cords where T lymphocytes can interact and establish cognate interactions with antigen-loaded dendritic cells and (3) a base, or nodal medulla, consisting of medullary cords, where plasma cell precursors derived from the GC response migrate and mature into antibody-secreting plasma cells [29].

Functional communication between the above described areas and the periphery is guaranteed by lymph-filled sinuses, interconnected structures surrounding lymphoid lobules: the subcapsular sinus associated with the afferent lymphatics at the LN apex (conveying dendritic cells from peripheral tissues to the paracortex), the transverse sinuses, and the paracortical-medullary sinuses ending with the efferent lymphatic vessel at the hilum (the output route of cells returning to the systemic circulation) [29]. The complementary path of LN cell trafficking takes place from the bloodstream through high endothelial venules (HEV), specialized vessels diffusely distributed within the nodal cortex, which allow naïve lymphocyte recirculation and local immune-surveillance through constitutive expression of addressins and homeostatic chemokines [30]. In the same LN area, a reticular network comprising reticular fibers, extracellular matrix components and stromal fibroblastic reticular cells (FRC) provides a three dimensional scaffold (the nodal conduit system) connected to both the parenchymal lymphatic apparatus and the peri-HEV space. This scaffold guarantees a close microanatomical connection between the nodal blood vascular apparatus and lymphatic network and the infrastructure for antigen and soluble substances transport within the LN parenchyma [30, 31].

## 3. Lymph Node Structural Changes during Experimental Immune-Inflammatory Challenge

Upon immune-inflammatory challenges, the homeostatic architecture and processes described in the previous section are subjected to modifications that lead to specific changes involving both the lymphocytic and the stromal component. These changes are collectively referred to as LN reactivity, a dynamic process involving induction and resolution phases, essentially aimed at the eradication of the triggers and return to homeostasis.

One of the most traceable pieces of evidence of reactivity is LN hypertrophy, the clinical sign and macroscopic outcome of a series of biological modifications, partly dependent on lymphotoxin *β* receptor (LT*β*R) signaling and the TNF ligand superfamily member 14 (TNFSF14/LIGHT) [32], inducing dramatic changes in local leukocyte trafficking, expansion of the fibroblastic stromal network [33, 34], and LN hyper-cellularity. The former changes include increased mobilization of mature dendritic cells from peripheral tissues through the afferent lymphatics, increased recruitment of lymphocytes from the bloodstream, and transient reduction of leukocyte egress from the efferent lymph, a phenomenon conventionally defined as LN “shut-down” [35]. Collectively, these events are instrumental in facilitating the encounter of antigen-loaded APCs with rare circulating antigen-specific lymphocytes as well as the intercellular cooperation between antigen-specific T and B cells, a process required for the initiation of the T cell dependent GC, one of the most evident signs of the ongoing adaptive response within a reactive LN [29].

Whilst changes in cellular input to the node are in part mediated by changes in HEV molecular activation [30], LN reactivity can also lead to the early and reversible modification of local blood flow supply and LN micro-angioarchitecture. Data supporting these concepts, which have prompted recent efforts aimed at tracking human LN reactivity through ultrasonographic assessments of local vascularity (see Section 5) [36], come from independent analyses performed over the last four decades demonstrating the effect of immune-inflammatory challenges on the enhancement of LN blood flow [37, 38] through remodeling of the feed arteriole [39], and on the upregulation of nodal angiogenesis [40, 41] through a mechanism involving FRC-derived vascular endothelial growth factor (VEGF) and CD11c+ dendritic cells [42, 43].

In parallel with the described modifications of the blood vascular system, the reactive LN can also undergo substantial changes in the local lymphatic apparatus and lymphatic drainage, processes that are now the object of growing attention in the field of chronic inflammatory diseases as potential check-points of peripheral pathology control (see Section 4). For decades the lymphatic network has been indeed considered as an inert drainage system. However, it is now clear that lymphatic vessels (both in peripheral tissues and in the LN) are actually highly dynamic structures, tightly regulated and actively implicated in the regulation of downstream immunological events. Fundamental studies elucidating the dynamic behavior of nodal lymphatic vessels, its mechanistic bases and immunological outcomes have been carried out by independent groups in recent years demonstrating the expansion of the LN lymphatic apparatus (with vessel proliferation) after local immunization [44], the coregulated expansion of LN blood/lymphatic vessels by the sequential activity of CD11c+ dendritic cells and lymphocytes [33], and the downstream enhancing effect of this process on local migration of dendritic cells [44]. As inferred by time-course analyses following LN experimental challenge in mice, both blood vessel proliferation and lymphatic system expansion in reactive LN have been shown to be spontaneously reversible* in vivo* [40, 45, 46], pointing at the existence of postreactivity regulatory steps, physiologically instrumental in the reestablishment of LN stroma homeostasis.

## 4. Lymph Node Involvement and Pathobiologic Role in Chronic Arthritis Models

Indubitably, full understanding of LN responses following experimental immunization represents an essential step to provide a rationale biologic platform for the development of LN research in humans. However, to what extent does experimental manipulation fully recapitulate the dynamics of LN challenge in chronic inflammatory settings remains the object of intense investigation. In particular, for what concerns RA, there are at least three issues that need to be taken into account: (1) the chronicity of the peripheral inflammatory process, which may last for months (at least at subclinical level), leading to potential exhaustion of LN response mechanisms acting in course of acute inflammation, (2) the lack of knowledge regarding the nature and anatomic distribution of the primary triggers of the dysregulated immunological response, whose activation may be ubiquitous or tissue-specific, transient or intermittent, unresponsive to conventional treatments or subjected to therapeutic interference, and (3) the unique pathologic features of the inflammatory process that can lead to massive hypertrophy of the target tissues with potential unbalance in draining LN response capacity during the progression of the disease. Due to the intrinsic difficulties and ethical limitations in assessing the functional and dynamic properties of the LN compartment in course of human disease, animal models of chronic (autoimmune) arthritis represent therefore a fundamental tool to advance mechanistic knowledge in the field.

The main aspects of RA pathology that could be supported by the activity or by a defective function of peripheral nodes can be summarized into three, nonmutually exclusive, points: (1) the local generation of autoimmune responses, (2) the maintenance of peripheral tolerance, and (3) the regulation of the peripheral inflammatory status through drainage of cells and fluids [10].

Compelling evidence supporting the possible contribution of the LN in the generation and perpetuation of arthritogenic autoimmunity has been provided by Mandik-Nayak et al. [47]. The authors carried out a systematic analysis of the autoimmune response in secondary lymphoid tissues of K/BxN mice, a murine model generated by crossing KRN T cell receptor transgenic (Tg) mice on a C57BL/6 background with NOD mice. K/BxN mice develop arthritis at 1 month of age and generate spontaneous responses against the ubiquitous autoantigen glucose-6-phosphate-isomerase (GPI). By tracking the anti-GPI B cell response in different anatomic sites at different stages of the disease (preclinical, acute, and chronic), the authors could demonstrate, in parallel with arthritis onset and appearance of serum autoantibodies, the localization of anti-GPI+ B cells (and differentiation of anti-GPI secreting B cells) within GCs and medullary cords of peripheral reactive LNs. Of note, despite ubiquitous autoantigen expression, the autoimmune response was shown to start specifically in the LN draining target joints, spreading thereafter to other lymphoid stations including the spleen. Alongside highlighting the early role of peripheral lymphoid tissues in a model of systemic arthritogenic autoimmunity, these data pointed out also at the potential relevance of the juxta-articular lymphoid stations in the pathogenic cascade of the inflammatory process.

In keeping with this concept, the dynamics and regulators of the joint-draining LN functional unit in course of arthritis have been the object of intense investigation in recent years, with increasing attention also on nonimmunological aspects. Several studies in this direction have been performed in the TNF-transgenic system, a* bona fide* adaptive immune response-independent model of arthritis, starting in the ankles, with subsequent heterogeneous involvement of the knees, preceded by dynamic changes in popliteal LN volume, blood flow, and lymphatic sinuses' size [48–50]. Despite the absence of detectable signs of B cell immune-reactivity, as inferred by the lack of activation-induced cytidine deaminase (AID) and Blimp-1 upregulation [51], initial evidence supporting a role of the draining LN in this system has been provided by Proulx et al. [49]. Through prospective, contrast-enhanced magnetic resonance imaging (CE-MRI) studies, the authors could demonstrate the significant negative correlation between popliteal LN size/lymphatic drainage capacity and progression of ipsilateral knee synovitis during the spontaneous course of the disease. Direct proof of these concepts has been subsequently obtained by gain-loss of function experiments in the same mouse strain showing, through VEGFR-3 blockade and VEGF-C intra-articular gene transfer, the protective effect of lymphatic drainage to the popliteal LN on local synovitis, bone erosions and cartilage loss [52, 53].

The observed heterogeneity of popliteal LN draining capacity, and its recognized pathophysiologic relevance during the spontaneous course of the disease in TNF transgenic mice, has led to further studies aimed at characterizing the cellular regulatory mechanisms of these processes. In this context, a relevant observation has been the identification of the potential antibody-independent role of nodal B cells [54] on the mechanical control of LN lymphatic flow. Evidence supporting this hypothesis derives from the work performed by Li et al. [51, 55] who, through time-course assessments of TNF-transgenic arthritis progression, could demonstrate the existence of an LN “collapse” phase, a postexpansion phase tightly coupled with worsening of arthritis in ipsilateral joints. LN “collapse” is characterized by LN volume contraction, disruption of nodal microarchitecture with massive B cell translocation into paracortical sinusoidal spaces, and reduction of local lymphatic drainage. Direct support to this mechanistic model and to its potential clinical relevance has been provided by response to treatment studies with B cell depleting agents, showing the efficacy of anti-CD20 monoclonal antibodies on the depletion of LN B cells, and its unexpected positive effect on the increase of LN lymphatic drainage with reduction in ipsilateral synovitis [51, 56].

To what extent (i) is the process of B-cell driven LN “collapse” a generalized feature of LN reactivity to immune-inflammatory challenge (see previous section) or is a specific response to chronic inflammation and (ii) whether these dynamics can be applied to human disease and treatment, still remain to be defined.

## 5. Evidence of Lymph Node Involvement in Patients with Rheumatoid Arthritis

Clinically, LN involvement in patients with RA is well established, and lymphadenopathy is consistently recognized among the possible extra-articular manifestations of the disease [57]. Since its first description accredited to Chauffard and Ramond in 1896, LN enlargement has been repeatedly confirmed in historical case series of the first half of the last century, with a frequency of detection ranging from 19% to 96% [58]. Despite the clinical interest in RA lymphadenopathy has diminished over the years due to the nonthreatening nature of the condition, earlier studies on the extra-articular features of RA report equally variable frequencies. Palpable LNs were detected in 41% of 102 British patients within the first 4-5 years from disease onset [59] and in 1.7% of 587 Italian RA patients with short disease duration (9.6 ± 8.6 months) [60]. The extreme variability reported in the literature could be attributed to several factors, including differences in the clinical features of the subjects analyzed, heterogeneous disease duration, geographic variations, and sites and modality of LN evaluation.

Histologically, lymphadenopathy associated with RA usually demonstrates reactive follicular hyperplasia and polyclonal plasma cell infiltration in the interfollicular area [61–63] as well as increased GCs with high B cell activity [64]. A moderate degree of vascular proliferation has also been reported [65]. Compared with nonrheumatoid follicular hyperplasia, the LNs in patients with active RA show increased numbers of CD8+ T lymphocytes in GCs and reduced IL-2R+ cells in the paracortex, possibly due to active recirculation of activated lymphocytes within the inflamed synovium [63]. Supporting that such morphological features may reflect active involvement of LN GCs in the generation of autoimmune responses typical of RA, LN areas occupied by follicles were described to correlate with rheumatoid factor (RF) status and titres [63]. More directly, Mellors et al. [66], by incubating RA axillary LNs with fluorescent agglutinated *γ*-globulins, reported the presence of RF in GC-localized cells and plasma cells in one out of ten GCs from a seropositive RA patient with active disease. These studies were confirmed 30 years later by Imai et al. [67], who demonstrated the presence of IgM RF in the LNs of seven out of seven seropositive patients but not in normal controls.

A critical question that remains unsolved is whether the LN compartment represents a primary site for the generation of initial autoimmune responses leading to RA. Incidentally, the occurrence of LN reactive hyperplasia before the onset of joint symptoms was already documented in historical studies performed in healthy subjects with unexplained lymphadenopathy [62]. More recently, the development of ultrasound-guided LN biopsy sampling procedures has considerably expanded the possibility of more systematic studies aimed at analyzing the early pathologic changes eventually occurring in healthy subjects at risk of developing RA [68]. In this direction, van Baarsen et al. [69] have investigated the cellular composition of inguinal LNs in individuals positive for RF and/or anticitrullinated protein antibodies (ACPA), in early arthritis patients and in autoantibody-negative healthy controls. The LN specimens were analyzed by multicolour flow cytometry to study different T and B lymphocyte subsets. In this exploratory study, the authors could recognize more CD19+ B cells and activated CD69+ CD8+ T cells in early arthritis patients and a trend toward increased CD19+ B cells in at risk subjects compared with controls. Together with the reciprocal low B cell signature already described in the peripheral blood [70], these findings have suggested the hypothesis that B cells may be retained within the LNs in the earliest phases of the disease [69].

As summarized in Section 4, recent studies in animal models of arthritis have highlighted the potential specific role of the juxta-articular LN stations in modulating the inflammatory cascade through immunological and nonimmunological functions. To what extent does the regional relationship between the lymphoid and the articular compartment through the afferent lymphatic system impact disease activity in humans is however unknown. Clinically, palpable LNs in patients with RA appear in anatomical relation with actively involved joints. Based on a clinical survey of 100 RA patients and 100 age- and sex-matched controls, Robertson et al. [58] reported a higher incidence of inguinal, axillary, and epitrochlear but not cervical lymphadenopathy in RA compared with controls, with mean joints scores of hands and wrists significantly higher in patients with palpable epitrochlear glands [58]. More recent studies have confirmed that, compared to systemic autoimmune diseases in which LN enlargement is more generalized, palpable LNs in active RA are more frequently located in the axillary area draining the hand joints, and clinical remission is paralleled by LNs disappearance/reduction [71]. Sporadic reports on positron emission tomography (PET) imaging in patients with active RA lend further support to clinical findings, confirming that LN uptake mostly corresponds to involved joints [72–74]. Furthermore, in keeping with MRI studies in animal models, the number and the size of draining LNs were shown to cross-sectionally correlate with the inflamed synovial volume at the knee through contrast enhanced three-dimensional-fast spoiled gradient echo (3D-FSPGR) MRI [75]. Although a more specific characterization of the cellular, molecular, and fluid recirculation across the articular and the LN compartments would require additional insights, the analysis of prenodal lymph from the foot of RA patients has revealed high lymph concentrations of cytokines and chemokines exceeding those in serum, supporting their local production within peripheral inflamed tissues [76]. Collectively, the above observations thus indicate that a reciprocal relationship between inflamed peripheral joints and the draining LNs can be also established in course of human arthritis.

In this context, a challenging issue still remains the full understanding of the temporal dynamics and anatomic distribution of (draining) LN involvement across the different phases of the disease, an objective that would require reliable and noninvasive methods of LN evaluation for serial and systemic analyses. Imaging studies performed in the oncology field have shown that power doppler ultrasonography (PD-US) allows sensitive assessment of the nodal structure and macrovessel architecture [77], which typically undergoes extensive remodeling upon immune-inflammatory stimulation (see Section 3). Using this approach, our group could recently show that subclinical changes such as vascular flow modulation and cortical hypertrophy are detectable in axillary LNs in patients with active RA, and that these parameters can be monitored prospectively in clinical settings [36]. The validation of reliable assessment techniques with noninvasive imaging approaches might open the possibility of comparative and multicompartment analysis of local disease activity in patients with RA.

## 6. Research Perspectives

**Figure 1:**
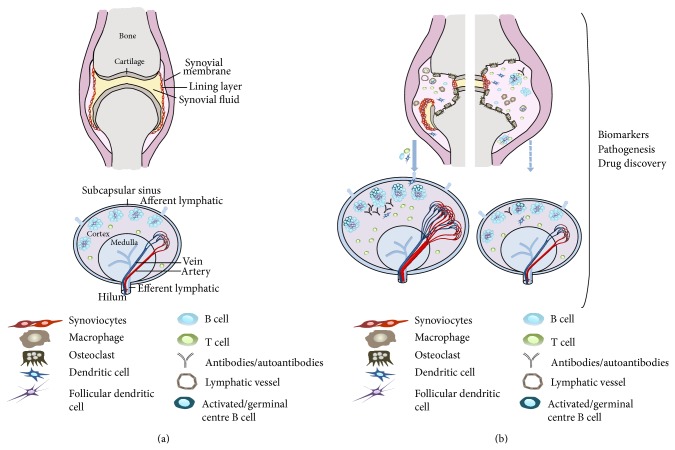
Potential pathophysiologic changes of and interactions between the joint and draining LN in course of inflammatory arthritis. (a) Schematic representation of the main anatomic compartments within a noninflamed joint and a resting LN. (b) Hypothetical spectrum of interactive relationships between the joint and the draining LN in course of arthritis. Joint inflammation promotes local accumulation of immune cells, synovial tissue hypertrophy and structural damage through the differentiation/activation of osteoclasts. These changes can promote increased fluid and cell drainage to adjoining LN through the afferent lymphatic system. An efficient drainage activity (left diagram, blue-filled arrow) may exert a compensatory effect on joint pathology by promoting fluid and cell exit. As an additional (nonmutually exclusive) effect, it might also contribute to disease immune-pathology by favouring neoantigen delivery, local immune-reactivity and (auto)antibody production. In the left LN diagram of (b), these mechanisms, together with putative structural changes (hypertrophy, cell accumulation, follicular hyperplasia, and increased vascularity) characterizing the challenged LN are shown. On the other side, a defective or insufficient drainage activity to the node (right diagram, blue-dashed arrow) may directly contribute to accumulation of inflammatory cells in the joint, promoting local cell activation and enhancing local disease (right hand side diagram of the joint).

The data presented in the previous sections of this review support the role of the LN as a potential relevant component of arthritis pathology, opening the attracting perspective of extending our research focus to this anatomic compartment as a complementary tool for the implementation of current prognostic biomarkers, the definition of preventive strategies, as well as the development of novel therapeutic approaches (Figure 1). The same data, however, also point at the significant gaps that still characterize current knowledge in the field with specific reference to the biology, functional dynamics, and hierarchical role of the LN compartment in human disease, both in its preclinical phases and in its established stage.

### 6.1. Autoimmunity and Peripheral Tolerance

In this regard, one of the most critical issues (from both a rheumatologic and immunological perspective) is represented by the actual contribution of the LN to the induction, maintenance, and perpetuation of the autoimmune response, with specific reference to the activation of ACPA-positive B lymphocytes, an RA-specific autoimmune trait related to a more severe course of the disease and contributing to the upstream phases of osteoclastogenesis [78, 79]. Despite the presence of B cells and plasma cells reacting against citrullinated peptides and being capable of spontaneous ACPA secretion* ex vivo* has been proved in the inflamed synovium of patients with established disease [80, 81], circulating ACPA are detectable years before the clinical onset of the disease [78], and their induction can take place before inflammatory changes in the joints have established [17]. This supports the concept that alternative/additional mechanisms and anatomic substrates may contribute to the induction/perpetuation of autoimmune responses during the pathologic history of RA [10, 82, 83]. Notwithstanding the potential role of the bone marrow [25, 28, 84] or the lung [85], both capable, as the synovium, of inflammation-driven ectopic lymphoid tissue formation, the physiological activity of secondary lymphoid tissues in the generation of adaptive immune responses clearly points at this compartment as a potentially critical site for both the pre- and the postarthritic ACPA production process. On the bases of these considerations, we may thus summarize current research questions in the field into three simple but critical points that still await to be convincingly addressed. (1) Do conventional T cell-dependent GC reactions within secondary lymphoid tissues participate in ACPA-positive B cell differentiation into ACPA-secreting plasma cells in course of RA? (2) Which is the relative contribution of secondary lymphoid tissues versus ectopic inflammatory sites in this process across progressive phases of the pathology (from pre-clinical to established disease)? (3) Does the LN draining affected joints play a primary role in the autoimmune response (as proposed in animal models, see Section 4) if compared to other (joint-unrelated) secondary lymphoid stations, such as the spleen, mesenteric LN, and other mucosal associated lymphoid tissues (MALT)?

Despite the assessment of these issues in humans still faceing considerable technical challenges, it certainly represents a critical target for our full comprehension of the disease and, hopefully, the conception of future therapeutic reprogramming strategies. In this perspective, it is important to emphasize that the LN not only acts as a site of construction of adaptive immune responses, but also represents a constitutive site of maintenance of peripheral tolerance. Data supporting this concept derive from different studies in mice and humans demonstrating the functional localization in nodal cells of the transcription factors autoimmune regulator (AIRE) and deformed epidermal autoregulatory factor-1 (DEAF-1), molecules that physiologically mediate autoreactive T cell deletion through the ectopic expression of a broad spectrum of peripheral tissue-restricted antigens (PTA) [86–89]. How these molecular systems operate in the context of autoimmune lymphoid tissues, whether they are involved in the process of tolerance disruption to citrullinated peptides and other RA-associated autoantigens, and whether they can be exploited for therapeutic purposes is currently unclear.

### 6.2. Biomarker Development

Beside the importance of understanding LN immunological behaviour, the same compartment may also be seen as a potential new source of biomarkers. Despite the introduction of biological agents and the optimization of clinical management (early diagnosis and treatment, tight control of disease activity) have considerably improved the outcomes and quality of life of patients with RA, the disease still presents a high degree of variability in terms of therapeutic response and radiographic progression. Validated tools able to fully predict this variability, likely determined by the intrinsic genetic and pathobiologic heterogeneity of the disease, are still missing, with consequent limitations in our possibility to define standard algorithms in critical stages of the disease, such as the selection of the appropriate approach in patients with early RA, the selection of the appropriate biological agent in patients nonresponding to conventional disease modifying antirheumatic drugs, and the identification of patients achieving pharmacologically induced clinical remission that can taper or suspend treatment with maintenance of health. Though the synovial tissue certainly represents the elective site for the definition of reliable indices embedded in the pathologic process [12–14], it is likely that future advancements in the field of biomarker discovery might benefit from progressing through the development of multiparameter modeling focused on both pharmacogenomic indices as well as on pathobiologic markers reflecting disease activity status in different compartments [13], including relevant juxta-articular sites. While the “juxta-articular perspective” has been intensively investigated for what concerns the subchondral bone marrow and the evaluation of bone marrow edema [27, 28], no data are currently available regarding the significance of draining LN assessment in large and well characterized clinical settings. As inferred by recent data in animal models and in humans, the draining LN could actually represent a relevant focus in this perspective, due to its variable involvement, its reactivity dynamics that may only partly overlap with joint inflammation, as well as due to its compensatory function through lymphatic drainage that could make it a complementary parameter for the integrated evaluation of local disease. Notwithstanding the obvious technical and ethical limitations in analyzing LN biology in large, consecutive and unbiased patient cohorts, a process that is required for biomarker validation in the human setting, there is now growing evidence (discussed in Section 5) that cellular [68, 69], structural, and functional [36] changes of the draining LNs can be potentially captured and monitored in RA. The implementation of repeatable imaging techniques able to sensitively identify these modifications with higher specificity and mini-invasive sampling approaches able to safely provide comprehensive biological insights represent therefore essential tasks for the translational development of LN research in human disease.

## 7. Conclusions

Our conception of RA as a disease with exclusive synovial extrinsication has greatly expanded over the past decades, with increasing awareness of the potential involvement of several articular and extra-articular districts along the time-line of RA development. Being the nerve centre for the generation of immune responses and the regulation of inflammatory processes, the lymphoid compartment comes to primary attention within the complex pathogenetic scenario of RA. Better understanding of the systemic and local LN responses might help clarifying the many uncertainties that still characterize the immunopathological features and clinical behaviour of chronic arthritis.
